# Clustering of Covid-19 morbidity cases in Germany

**DOI:** 10.1088/1757-899X/862/4/042037

**Published:** 2020-05

**Authors:** D A Petrusevich

**Affiliations:** Russian Technological University (MIREA), Prospekt Vernadskogo, 78, Moscow, 119454, Russiapetrdenis@mail.ru

## Abstract

The Covid-19 coronavirus has spread almost all over the world. Though it has been reported recently that the epidemic declines in China, in other countries it still hasn’t achieved peak level. The data analysis methods may help struggling against the disease. The Covid-19 Tracking Germany dataset has been handled in the research. It’s daily refreshed dataset available at the kaggle.com site. It contains information on number of fallen ill people in Germany. The cases are grouped by federal land, city, age diapason and date. The main goal of the research is to underline differences in morbidity registered in different lands of Germany. There have been published new suggestions about connection between coronavirus morbidity and BCG vaccination. This question is also taken into account. Analysis based on the handled dataset is able to make only oblique conclusions because of lack of information. Differences in coronavirus morbidity in various regions and various age groups are highlighted. The regions of Germany are clustered into groups by gravity of recent situation.

## 1. Introduction
In this research the data analysis methods are used in order to detect special features in morbidity and mortality of the coronavirus disease in Germany [1]. Nowadays data science and information technologies in common are used in a lot of tasks of various themes [2]. Today there are attempts to help medic staff understand special features of this disease. The federal lands of Germany are investigated because there are assumptions on the influence of BCG vaccines [3]. According to this publication, the vaccinated people have got light forms of the illness. Still there are debates on this question. Attempt to test this hypothesis has been done. The BCG vaccine implementation has been cancelled in Germany nowadays [4] but in the Eastern Germany this vaccine was implemented totally. If this hypothesis is true there should be statistical differences in data of the former Eastern Germany lands and the Western Germany regions. These features are investigated in the present paper.

Also division of federal lands into clusters by severity of the coronavirus is done. Nowadays this analysis is processed in a lot of projects of publications [5, 6]. It's necessary in order to understand special features in morbidity, make conclusions about differences of immune system structure of infected in light and heavy forms. Differences in age, population density in region, number of deaths and infection cases are analyzed.

## 2. The dataset structure
The Covid-19 Tracking Germany dataset [1] contains information on disease cases that is daily refreshed. There are columns: "state" containing name of the federal land where the cases have occurred [7], "county" holding information on the city or other administrative element where they have taken

Content from this work may be used under the terms of the Creative Commons Attribution 3.0 licence. Any further distribution of this work must maintain attribution to the author(s) and the title of the work, journal citation and DOI.
Published under licence by IOP Publishing Ltd place, “age-group”, “gender”, “date” containing date of the cases, “cases” and “deaths” holding information on number of illness cases and death cases. Information is grouped by date, federal land, city, age groups and gender.

According to [7] the federal lands list has been handled. Germany has got complicated history and administrative division. Some administrative elements have been united into groups: Saarland is the region transferred from France after the 2nd World War. This relatively small land by population has been combined with neighbouring Rhineland-Palatinate in the experiments.

There are cities that are still individual administrative elements. In this research they are combined with surrounding federal lands. Bremen has been combined with Lower Saxony, Schleswig-Holstein and Hamburg have been united.

There are few cities with population more than 1 mln. people. But in Berlin there are more than 3 mln. citizens [7]. This city is usually handled individually in the experiments.

Illness cases are grouped by age into categories: 0 – 4, 5 – 14, 15 – 34, 35 – 59, 60 years and older. The date of the handled dataset is the 2nd of April. It’s refreshed daily. Version of this day is used in the experiments. The last cases included into it are marked with the 1st of April. The first cases have been detected in Bayern on the 28th of January.

By the 2nd of April the number of coronavirus cases in Germany is 77477.

## 3. Experiments

The dataset [1] has been clustered manually by the illness spread rates and values of morbidity available in the data. Automatic methods of clustering including hierarchical divisive and agglomerative clustering [8, 9] are implemented in the second part of the experiment. Groups of regions with different behaviour of illness spread and morbidity are constructed and analyzed.

### *3.1. Division by regions*

The first parameter that should be mentioned is growth rate of illness cases. By this value the lands of Germany can be divided into three clusters. The first group with “low” speed of growth mainly include the lands of former Eastern Germany. The ratio of “deaths”/”cases” is less or equal to 1%, the maximum growth per day isn’t more than 200 person per day. For the majority of these lands the first case of illness has been detected much later than in the other groups. This cluster includes Saxony, Saxony-Anhalt, Mecklenburg-Vorpommern, Brandenburg, Thuringia, Saarland.

The second cluster of intermediate growth rate of illness cases number. The maximum growth doesn’t achieve 1000 person per day but it’s higher than in the first cluster. The ratio “deaths”/”cases” is approximately the same as in the first cluster. It includes Berlin, Rhineland-Palatinate, Hesse, Schleswig-Holstein (here united with Hamburg), Lower Saxony (here united with Bremen).

The third cluster includes regions with high growth rate of illness cases quantity. The ratio “deaths”/”cases” varies. Maximum speed of growth is more than 1000 person per day. The first cases are detected much earlier than in the other clusters. The “deaths”/”cases” is more than 1% and usually is twice more than in the first cluster. There are North Rhine-Westphalia, Bavaria (here the first cases in Germany have been detected), Baden-Wurttemberg.

Examples of growth rates in each cluster are presented at the figures 1 – 3. The x axis denotes days from the first case in the land. One can notice that during the first month there haven’t been a lot of illness cases in Bavaria (figure 3). The same is also right for Rhineland-Palatinate region: during the first ten days there’s no quick growth. The first case in Saxony has been noticed on the 2nd of March. The first case in Rhineland-Palatinate has been noticed on the 28th of February. The first case of illness in Bavaria has been noticed on the 28th of January.

IOP Conf. Series: Materials Science and Engineering **862** (2020) 042037 doi:10.1088/1757-899X/862/4/042037

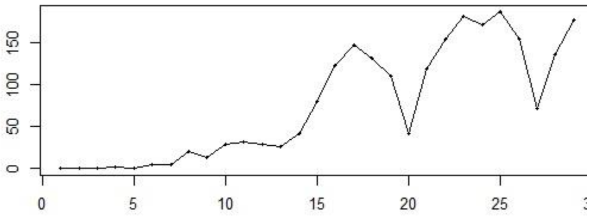

**Figure 1.** Dependence of daily growth rate in Saxony on time (the 1st day is the 2nd of March).

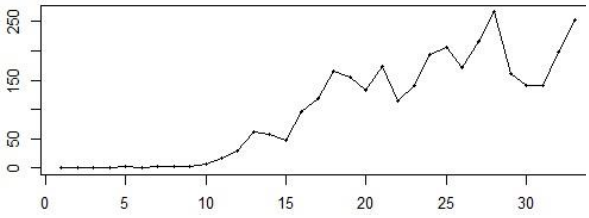

**Figure 2.** Dependence of daily growth rate in Rhineland-Palatinate on time (the 1st day is the 28th of February).

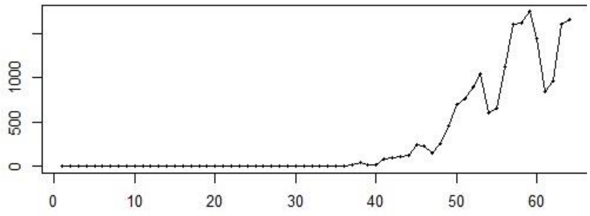

**Figure 3.** Dependence of daily growth rate in Bavaria on time (the 1st day is the 28th of January).

One can notice that growth rates have been at low level in the very beginning of the epidemic in all cases and they have started raising quickly approximately at 10th – 15th of March.

The majority of death cases is in the group of 60-99 years by age.

Statistical data that can be obtained from the dataset is presented in the table 1. The date when the first coronavirus case has been detected, the date when 50% of overall level infection (the 2nd of April is the date of the analyzed dataset) has been achieved are shown in the last columns. They can demonstrate how fast the growth rates increase. Population density of regions is taken from the statistical data of 2018 year [7].

**Table 1**. The statistical measures of illness spread in the lands of Germany.

| Region of Germany | Quantity of illness cases | Quantity of death cases | Percent of “deaths” /”cases” ratio | Maximal growth rate per day | Population density (person / km$^2$) | Date of the first illness case | Date of 50% level |
|---|---|---|---|---|---|---|---|
| Mecklenburg-Vorpommern | 424 | 4 | 0.9 | 49 | 69.1 | 2020-03-03 | 2020-03-24 |
| Saxony-Anhalt | 769 | 11 | 1.4 | 78 | 108 | 2020-03-10 | 2020-03-24 |
| Thuringia | 885 | 9 | 1.0 | 83 | 132.3 | 2020-03-03 | 2020-03-24 |
| Brandenburg | 1020 | 9 | 0.9 | 112 | 84.71 | 2020-03-01 | 2020-03-24 |
| Saxony | 2178 | 21 | 1.0 | 186 | 221 | 2020-03-02 | 2020-03-24 |
| Berlin | 2837 | 20 | 0.7 | 280 | 4090 | 2020-03-03 | 2020-03-24 |
| Hesse | 3876 | 33 | 0.9 | 310 | 296.7 | 2020-02-28 | 2020-03-24 |
| Schleswig-Holstein and Hamburg | 3899 | 31 | 0.8 | 277 | 286.2 | 2020-02-28 | 2020-03-24 |
| Rhineland-Palatinate and Saarland | 4324 | 37 | 0.9 | 369 | 226.3 | 2020-02-28 | 2020-03-25 |

| | | | | | | | |
|---|---|---|---|---|---|---|---|
| Lower Saxony and Bremen | 4659 | 65 | 1.6 | 397 | 180.0 | 2020-02-04 | 2020-03-24 |
| Baden-Wurttemberg | 14581 | 54 | 1.9 | 1195 | 309.7 | 2020-01-30 | 2020-03-24 |
| North Rhine-Westfalia | 15620 | 176 | 1.1 | 1112 | 525.7 | 2020-02-22 | 2020-03-24 |
| Bavaria | 19205 | 304 | 1.6 | 1748 | 185.4 | 2020-01-28 | 2020-03-29 |

One can notice that there's high quantity of illness cases in the lands with the maximal density of population. Though the density is lower in Bavaria. At the same time these are the lands that are close or have got borders with countries in which situation is very difficult (Netherlands and Italy). The highest growth rate is marked in Bavaria.

The lands of the second cluster: Hesse, Schleswig-Holstein and Hamburg, Rhineland-Palatinate and Saarland, Lower Saxony and Bremen have got lower values of ratio "deaths"/"cases". Berlin has got very high density and still situation there is better than in the third cluster. Lower Saxony and Bremen have got lower density but the ratio is high.

The lands of the first cluster have got low density of population. Though their ratio values are higher than ones in the second cluster.

Almost in all lands 50% of infected people rate (data of the 2nd of April) has been achieved on the 23rd of March. Thus approximately during one week number of infected people doubled. It means that the peak of morbidity is still in the future.

One can conclude that by absolute numbers the former Eastern Germany regions handle the coronavirus epidemic better than other regions. At the same time looking at the relative parameters one can see that the situation is slightly better in Schleswig-Holstein (here with Hamburg) and in Hesse, Rhineland-Palatinate, Saarland regions.

Looking at figures 1 – 3 one can see that the epidemic still doesn't achieve its peak values.

### *3.2. Influence of BCG vaccine implementation on coronavirus infection rates*

New research on BCG application [3] is devoted to statistical comparison between coronavirus infection rates, its severity and rates of BCG vaccines implementation. Available statistics isn't enough to make thorough research and conclusions. Information in the handled dataset can be analyzed to make tests on this hypothesis. Nowadays the BCG overall implementation is cancelled in some European countries including Germany [4]. But in the former Eastern Germany this procedure was done. Thus one can try to find statistical traces of this notice. There are two problems that can't be investigated observing ordinary open data. The first one is "expiration date" of BCG vaccine. It produces complex effect on organism and can be used to a set of illnesses, not only against the tuberculosis. But this effect weakens with time. The individuals younger than some limit domain of age should go through coronavirus easier. At the same time the deaths rate is higher for the group people of 60-99 years. The vaccine has got limited effect according to this hypothesis [3]. The second problem is lack of statistical data on people of former Eastern Germany. A lot of them have migrated to the lands of the Western Germany. And they participate in the statistics of these regions. At the same time one have to propose that the majority of people older than 30 years living in the federal lands of former Eastern Germany are born there and have been vaccinated.

The first idea to be tested is the comparison of regions that were included into the Eastern and Western Germany on the number of deaths. This information is shown in the table 2. Three age groups have been analyzed: 15-34 years, 35-59 years and older than 59 years. Quantity of illness cases, deaths are presented in the first column in each pair. The ratio "deaths"/"cases" is shown in the second column in each pair.

**Table 2**. The coronavirus mortality rates in the lands of Germany.

| Region of Germany | Quantity of death cases / illness cases (age: 15-34) | Percent of death cases / illness cases ratio (age: 15-34) | Quantity of death cases / illness cases (age: 35-59) | Percent of death cases / illness cases ratio (age: 35-59) | Quantity of death cases / illness cases (age >60) | Percent of death cases / illness cases ratio (age > 60) |
|---|---|---|---|---|---|---|
| Mecklenburg-Vorpommern | 0 / 132 | 0 | 1 / 187 | 0.5 | 3 / 91 | 3.2 |
| Saxony-Anhalt | 0 / 180 | 0 | 0 / 356 | 0 | 11 / 214 | 5.2 |
| Thuringia | 0 / 213 | 0 | 1 / 429 | 0.2 | 8 / 219 | 3.7 |
| Brandenburg | 0 / 230 | 0 | 0 / 517 | 0 | 9 / 255 | 3.5 |
| Saxony | 0 / 525 | 0 | 1 / 1027 | <0.1 | 20 / 563 | 3.6 |
| Berlin | 0 / 1010 | 0 | 3 / 1269 | 0.2 | 17 / 440 | 3.9 |
| Hesse | 0 / 894 | 0 | 1 / 1989 | <0.1 | 32 / 923 | 3.5 |
| Schleswig-Holstein and Hamburg | 0 / 1134 | 0 | 3 / 1741 | 0.1 | 28 / 873 | 3.2 |
| Rhineland-Palatinate and Saarland | 0 / 1120 | 0 | 4 / 2090 | 0.2 | 33 / 1012 | 3.3 |
| Lower Saxony and Bremen | 0 / 1220 | 0 | 5 / 2322 | 0.2 | 66 / 1613 | 5.0 |
| Baden-Wurttemberg | 0/3639 | 0 | 11/6861 | 0.2 | 269/3940 | 6.8 |
| North Rhine-Westfalia | 0 / 3828 | 0 | 9 / 7747 | 0.1 | 167/3722 | 4.5 |
| Bavaria | 2 / 4865 | <0.1 | 15 / 8540 | 0.2 | 286/5042 | 5.7 |

The first column is 0 almost for all regions: young people don't die because of the coronavirus. There are some death cases if an age is between 15 and 34 years old. These cases should be handled individually but there's no open data. One can suppose that there have been chronic health conditions [5, 6]. And at last percent of deceased people older than 59 years is in the interval 3 – 7%. One cannot say that the Eastern Germany regions have got lower ratio. There are the highest percents in the lands: Bavaria, Baden-Wurttemberg, Lower Saxony (with Bremen), North Rhine-Westfalia and Saxony-Anhalt (though absolute numbers in this region are extremely low).

The hypothesis about the BCG influence isn't confirmed here. But as it was mentioned above there isn't enough information in the dataset to test this idea. At the same time it's possible to check the density of the infected people. The population density varies in different lands. So, it would be better not to divide number of cases on population of a land but to use population density values. If the quantity of infected people increases this value also grows. If the density grows (the population grows but area is the same) the ratio decreases. Results of this experiment are presented in the table 3.

**Table 3**. The statistical measures of illness spread in the lands of Germany.

| Region of Germany | Quantity of illness cases | Population density (person / km$^2$) | Illness cases / population density ratio |
|---|---|---|---|
| Mecklenburg-Vorpommern | 424 | 69.1 | 6.14 |
| Saxony-Anhalt | 769 | 108 | 7.12 |
| Thuringia | 885 | 132.3 | 6.69 |
| Brandenburg | 1020 | 84.71 | 12.04 |
| Saxony | 2178 | 221 | 9.86 |

| | | | |
|---|---|---|---|
| Berlin | 2837 | 4090 | 0.69 |
| Hesse | 3876 | 296.7 | 13.06 |
| Schleswig-Holstein and Hamburg | 3899 | 286.2 | 13.62 |
| Rhineland-Palatinate and Saarland | 4324 | 226.3 | 19.11 |
| Lower Saxony and Bremen | 4659 | 180.0 | 25.88 |
| Baden-Wurttemberg | 14581 | 309.7 | 47.08 |
| North Rhine-Westfalia | 15620 | 525.7 | 29.71 |
| Bavaria | 19205 | 185.4 | 103.59 |

Values in the last column of the table 3 don't depend on the population density or area of lands. In this experiment the lands can also be separated into three clusters that have got much in common with the differentiation in the paragraph 3.1. The former Eastern Germany without Brandenburg (Mecklenburg-Vorpommern, Saxony-Anhalt, Thuringia, Saxony and even Berlin which should be handled separately) has got ratio less than 10.

The intermediate cluster contains Brandenburg separated from the cluster of the former Eastern Germany and the following lands: Schleswig-Holstein and Hamburg, Rhineland-Palatinate and Saarland, Hesse, North Rhine-Westfalia, Lower Saxony and Bremen. The ratio is less than 30 in this group. North Rhine-Westfalia has got the highest population density. Because of that the ratio isn't very high though absolute values can be interpreted as a disaster.

The regions with heavy coronavirus situation are Bavaria and Baden-Wurttemberg.

### *3.3. Automatic clustering*

After the step of analysis based on perception of the dataset is over it's possible to construct new dataset with results of two previous sections. The first experiment handles new dataset that is constructed of the columns of the table 1 except dates. Also number of infected people in categories of 15 – 34, 35 – 59, 60 years old and older; deaths in categories of 35 – 59, 60 years old and older; the ratio of deaths / infected people of 60 years old and older are included into new dataset. Each column in this dataset is scaled according to expression (1):

$$x' = \frac{x-\mu}{\sigma}. \quad (1)$$

Here x is an old value, $x'$ is the transformed one, $\mu$ is its mean value and $\sigma$ is its standard deviation. After this step standard deviation of all the values is 1, mean value is 0.

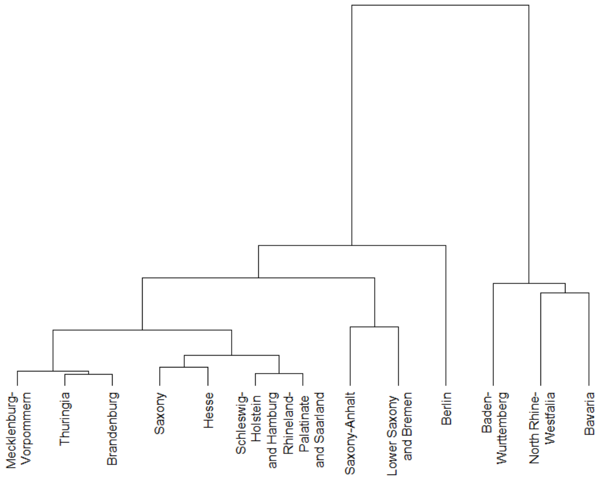

**Figure 4.** Agglomerative clustering of German lands by coronavirus morbidity (five clusters).

Agglomerative clustering method has been implemented to unite lands of Germany into new groups (clusters). The Euclidean metrics has been used to measure distance between classes and the Ward

IOP Conf. Series: Materials Science and Engineering **862** (2020) 042037 doi:10.1088/1757-899X/862/4/042037

method has shown the best results in order to combine them (the agnes command is used). The dendrogram is presented in figure 4.

One can notice that regions with heavy situation are combined into one cluster: Bavaria, North Rhine-Westfalia and Baden-Wurttemberg. Berlin is separated into its own cluster because of its high population (in comparison to the other regions of Germany). Saxony-Anhalt and Lower Saxony (with Bremen) are united into the third cluster (they've got close values of deaths / infected people ratio presented in the table 1). The lands of the fourth cluster are also close by the same parameter: Saxony, Hesse, Schleswig-Holstein (with Hamburg) and Rhineland-Palatinate (with Saarland) have got deaths / infected people ratio close to 3%. Regions of Mecklenburg-Vorpommern, Brandenburg and Thuringia are united into the fifth cluster. It should be mentioned that these regions were parts of the former Eastern Germany. Other lands are included into the third and fourth clusters. Berlin is a separated cluster as it was mentioned above. Here one can't conclude that there are differences between former Western and Eastern Germanies. The agglomerative coefficient is about 87%.

The second clustering experiment handles dataset containing number of overall illness cases, population density and result of their division (the table 3). The divisive hierarchical clustering method (the diana command is used) has shown the best results. The divisive coefficient is about 88% [8, 9].

The first cluster contains all lands of the former Eastern Germany except Berlin; Berlin is separated into an individual cluster; the third cluster contains Hesse, Schleswig-Holstein (with Hamburg), Rhineland-Palatinate (with Saarland) and Lower Saxony (with Bremen). The fourth cluster contains the regions with maximal quantity of infected people: Bavaria, North Rhine-Westfalia and Baden-Wurttemberg.

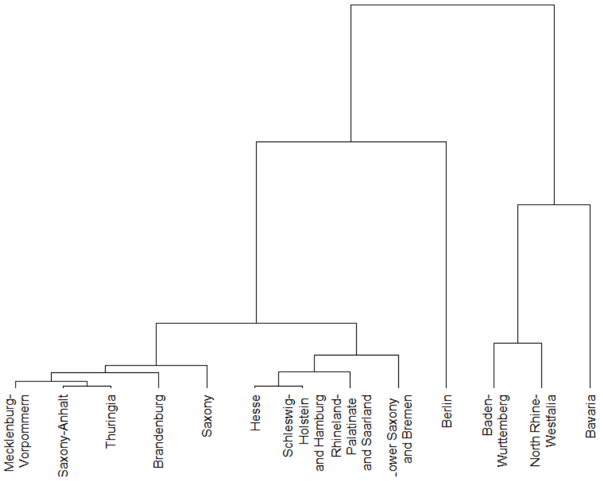

**Figure 5.** Divisive clustering of German lands by coronavirus morbidity (four clusters).

Thus it's possible to conclude that the former Eastern Germany lands behave in a different way during the epidemic. But the thorough analysis has to use individual depersonalized information on implementation of vaccines. Also other clustering techniques (for example, [9]) are going to be implemented in further research.

## 4. Conclusion

The coronavirus in Germany dataset [1] has been investigated in this paper. The clustering of the German regions by severeness of the illness has been done. There are regions with heavy situation (Bavaria, North Rhine-Westfalia and Baden-Wurttemberg), lands with low speed of spread (Mecklenburg-Vorpommern, Brandenburg, Thuringia) and regions with intermediate speed (Hesse, Schleswig-Holstein and Hamburg, Rhineland-Palatinate and Saarland, Lower Saxony and Bremen).

The automatic agglomerative and divisive clustering methods have got common results and there are some differences. The cluster of regions with very high speed of infection spread appears in both experiments. Berlin is separated into an individual cluster because it's very large city by population in comparison with other lands of Germany. Other clusters contain lands with low and intermediate speed of coronavirus spread.

Also the hypothesis on BCG vaccine influence on the morbidity and mortality from the coronavirus [3] has been investigated. The vaccine has been implemented to all citizens of the former Eastern Germany. At the same time in the Western Germany the total vaccination hasn't been done. The BCG overall implementation is cancelled in some European countries including Germany. The hypothesis could be tested on the data from Germany. Thus there should be statistical differences in the morbidity in various regions of Germany. There are such features and in one of the clustering experiments all the lands of the former Eastern Germany have been included in one cluster of low speed of illness spread (except Berlin). But to make confident conclusions one has to use medical data and information about people who moved between regions of former western and eastern parts of Germany. Thus, further analysis is required and datasets of BCG vaccine implementation is necessary to complete such research.
